# Enhancing clinical utility: deep learning-based embryo scoring model for non-invasive aneuploidy prediction

**DOI:** 10.1186/s12958-024-01230-w

**Published:** 2024-05-22

**Authors:** Bing-Xin Ma, Guang-Nian Zhao, Zhi-Fei Yi, Yong-Le Yang, Lei Jin, Bo Huang

**Affiliations:** 1 Reproductive Medicine Center, Tongji Hospital, Tongji Medical College, Huazhong University of Science and Technology, Wuhan, 430030 China; 2 Department of Obstetrics and Gynecology, National Clinical Research Center for Obstetrics and Gynecology, Tongji Hospital, Tongji Medical College, Huazhong University of Science and Technology, Wuhan, 430030 China; 3 Key Laboratory of Cancer Invasion and Metastasis (Ministry of Education), Hubei Key Laboratory of Tumor Invasion and Metastasis, Tongji Hospital, Tongji Medical College, Huazhong University of Science and Technology, Wuhan, 430030 China

**Keywords:** iDAScore, Time-lapse system, Embryo ploidy, Artificial intelligence

## Abstract

**Background:**

The best method for selecting embryos ploidy is preimplantation genetic testing for aneuploidies (PGT-A). However, it takes more labour, money, and experience. As such, more approachable, non- invasive techniques were still needed. Analyses driven by artificial intelligence have been presented recently to automate and objectify picture assessments.

**Methods:**

In present retrospective study, a total of 3448 biopsied blastocysts from 979 Time-lapse (TL)-PGT cycles were retrospectively analyzed. The “intelligent data analysis (iDA) Score” as a deep learning algorithm was used in TL incubators and assigned each blastocyst with a score between 1.0 and 9.9.

**Results:**

Significant differences were observed in iDAScore among blastocysts with different ploidy. Additionally, multivariate logistic regression analysis showed that higher scores were significantly correlated with euploidy (*p* < 0.001). The Area Under the Curve (AUC) of iDAScore alone for predicting euploidy embryo is 0.612, but rose to 0.688 by adding clinical and embryonic characteristics.

**Conclusions:**

This study provided additional information to strengthen the clinical applicability of iDAScore. This may provide a non-invasive and inexpensive alternative for patients who have no available blastocyst for biopsy or who are economically disadvantaged. However, the accuracy of embryo ploidy is still dependent on the results of next-generation sequencing technology (NGS) analysis.

## Background

Despite the notable advancements in assisted reproductive technology, the average live birth rate in the UK is still low, at 32% per embryo transfer (for women under 35 years old), therefore, the selection of the best embryo for transfer remains the fundamental difficulty in in vitro fertilization (IVF) field [1]. Aneuploidy is the primary cause of implantation failure and pregnancy loss [2]. Preimplantation genetic testing for aneuploidy (PGT-A), which allows the accurate analysis of all 24 chromosomes, has made it one of the best current technologies for choosing euploidy embryos [3]. However, its invasive nature due to the requirement for embryo biopsy might not be available because it is illegal or they could think it is unethical for certain people [2]. Or, they could also lack biopsy-ready embryos. Further restricting accessibility is the fact that PGT-A can cost up to $12 000 in the USA and over £3000 in the UK [4]. Additionally, improvements in PGT-A’s clinical result seem to only apply to women older than 37 years [5]. Therefore, the necessity for more non-invasively or sophisticated techniques to identify aneuploidy embryo is critical.

The introduction of Time-lapse (TL) in IVF field has provided ways to avoid some of the drawbacks of conventional morphological evaluation. It reduced the possible effects of fluctuations in temperature or gas composition, enabled continuous monitoring of embryo development, which improves our understanding of embryokinetics [6]. Therefore, several groups aimed to clarify the relationship between morphokinetic parameters got from TL and ploidy status. Some abnormal cleavage pattern was investigated, such as the degree of fragmentation, existence of direct and reverse cleavage, blastocyst contractions, and multinucleation etc [7–12]. These morphological features might help to distinguish ploidy types.

Theoretically, the artificial intelligence (AI)-powered TL evaluation represented a wealth of data that may be utilized for euploidy embryo selection [9, 13–15]. However, its practical applicability has to be evaluated in more well-designed research and/or sizable datasets. EmbryoScope + incubators could be connected to the software “intelligent data analysis (iDA) Score”. The deep learning algorithm used in this program, which was trained on hundreds of thousands of videos, assigned each embryo with a score between 1.0 and 9.9.

In present study, we aim to investigate the correlations between morphology and ploidy status. In particular, it is also determined whether the ability of iDAScore to discriminate embryo ploidy could be enhanced by incorporating some clinical features.

## Methods

### Study design

**Fig. 1:**
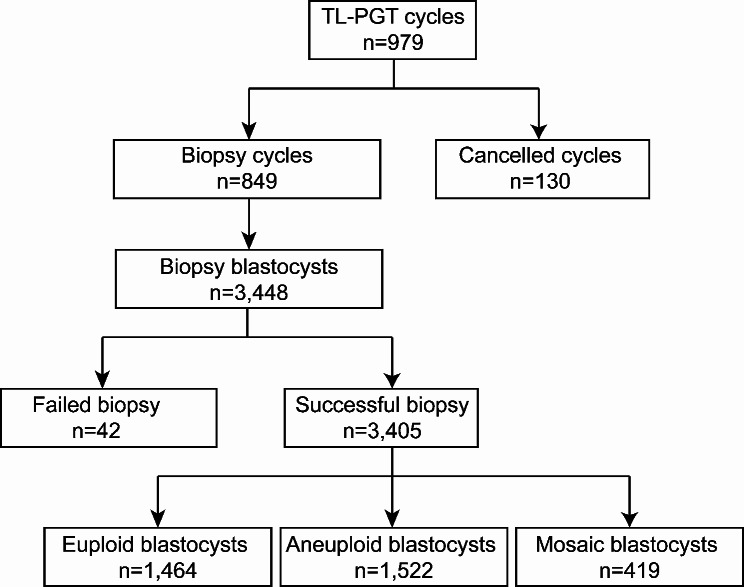
Schematic presentation of the study design. TL, time-lapse; PGT, preimplantation genetic testing

The retrospective cohort study involved 979 TL-PGT cycles conducted from 2018 to 2021 at the Reproductive Medicine Center, Tongji Hospital, Tongji Medical College, Huazhong University of Science and Technology, Wuhan, Hubei, China. A total of 3448 blastocysts were biopsied. These samples were lysed, and the DNA was fragmented and amplified. Among them, 3405 samples were amplified successful, and 42 samples were failed to amplify. In this work, the iDAScore model was used to retrospectively examine 3405 blastocysts that were cultivated in an EmbryoScope Plus (Vitrolife A/S, Denmark) incubator. In Fig. 1, the research design is displayed. On an informed consent form, each patient signed. Every patient signed an informed consent form. The study complied with the Declaration of Helsinki for Human Subjects in Medical Research and the Board of Institutional Review (No. 2019s097) approval was given by the Ethical Committee of Reproductive Medicine Center, Tongji Hospital, Tongji Medicine College, Huazhong University of Science and Technology.

### Ovarian stimulation and oocyte retrieval

Controlled ovarian stimulation (COS) was performed in compliance with our previous studies [16]. Patients were regularly monitored with transvaginal ultrasonography during COS. When the leading follicle(s) measured more than 18 mm, human chorionic gonadotropin (HCG) was administered. The oocytes were retrieved 36 h after the HCG injection, guided by ultrasonography. The collection of cumulus-oocyte complexes (COCs) was performed as described in our previous studies [17].

### Embryo culture and biopsy

The density gradient centrifugation method was used to optimize the semen samples [18]. The sperm concentration, motility, and morphology were evaluated using the fifth edition of the World Health Organization recommendations. During intracytoplasmic sperm injection (ICSI), the COCs were denuded two hours after retrieval, and sperm was injected four hours later. The resultant zygotes were subsequently cultured utilizing a time-lapse incubation system at G1 Plus (Vitrolife, Sweden). Each embryo was photographed every ten minutes in TL incubator. After insemination, pronuclei were inspected 16–18 h later. The culture medium was switched to G2 Plus (Vitrolife, Sweden) on the third day. The blastocysts that met the Gardner criterion (better than 3BC) were biopsied and then cryopreserved for later use on the fifth and sixth day. Rarely, the embryo was cultivated for vitrification up until the seventh day. Before performing a biopsy, a tiny hole in the zona pellucida is created using a laser. This allowed for the mechanical dissection of three to six trophectoderm cells.

### Next-generation sequencing technology (NGS) analysis

The PGT cycles were conducted with NGS analysis [19]. To summarize, the samples were amplified using a single-cell whole-genome amplification (WGA) based on multiple annealing and looping-based amplification cycles (MALBACs), in accordance with commercial kit protocol (Yikon Genomics). DNA was fragmented, amplified, labelled, and purified in a sequential manner. Utilizing Life Technologies’ Ion Proton technology, the final library was sequenced at a depth of around 0.04× genomes. In order to detect variants, this sequencing speed generates repeatable copy number variations (CNVs) at ∼ 4 MB resolution. A threshold of more than 70% was established for the detection of aneuploidy. When it comes to chromosomes, the threshold for mosaic detection differs. The lower limit was 30% for chromosomes 13, 16, 18, and 21, 50% for chromosome 19, and 40% for all other chromosomes. A number that is below the lower bound denotes euploidy.

### iDAScore analysis

A deep learning neural network that is trained on TL videos to predict fetal heartbeat is the iDAScore embryo scoring model. Time-lapse videos are fed into the iDAScore model, which generates an embryo score between 1.0 and 9.9. The data from the blastocysts that were included in this study were assessed retrospectively using the iDAScore model.

### Statistical analysis

Continuous variables with normal distributions were expressed as mean ± SD. Categorical variables were expressed as number and percentage (%). For data with a normal distribution, one-way analysis of variance (ANOVA) was applied for multiple comparisons. The chi-square test was used to compare categorical variables between groups. Multivariable logistic regression was applied to evaluate the association between the iDAScores and ploidy, and the odds ratios (ORs) were calculated. All statistical tests were two-sided and *p* values less than 0.05 were considered statistically significant. All analyses were conducted in SPSS Statistics (version 23.0, IBM, Armonk, NY, USA).

## Results

### Clinical characteristics of PGT cycles

A total of 979 TL-PGT cycles were involved in this present study. As shown in Table 1, the average maternal age is 31.78 and the average infertility duration is 2.07 years. Measurements for basic follicle-stimulating hormone (FSH), anti-Mullerian hormone (AMH), and body mass index (BMI) of females are also present. The proportion of different sperm quality among all cycles was calculated. Additionally, gonadotropin (Gn) dosage and duration are also described in Table 1.

**Table 1:** Clinical characteristics of PGT cycles

Parameter	
No. of cycles	979
Maternal age (y)	31.78 ± 4.44
Duration of infertility (y)	2.07 ± 2.22
Basic FSH (IU/L)	7.59 ± 2.75
Basic AMH (ng/mL)	4.42 ± 3.34
BMI	22.46 ± 7.19
Cycle with normal-quality sperm (%)	80.90 (792/979)
Cycle with poor-quality sperm (%)	17.67 (173/979)
Cycle with azoospermia (%)	1.43 (14/979)
Gn dosage (IU)	2547 ± 887
Gn duration (day)	9.82 ± 1.77
No. of oocytes	13,720
No. of matured oocytes	11,023
No. of two pronucleus	8114
No. of available blastocyst	3778

FSH, follicle-stimulating hormone; AMH, anti-Mullerian hormone; BMI, body mass index; Gn, gonadotropin

### The iDAScore of blastocysts with different ploidy

**Fig. 2:**
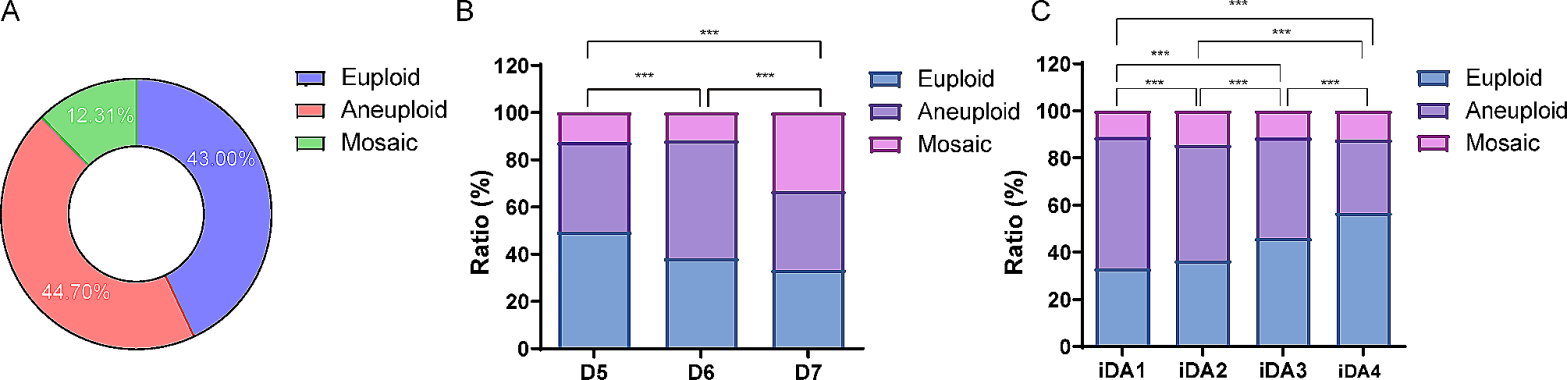
The ratio of blastocysts with different ploidy. (**A**) The ploidy of all blastocysts. (**B**) The ploidy of blastocysts with different length of incubation. (**C**) The ploidy of blastocysts with different iDAScores. *** presents *p* < 0.001

As shown in Fig. 2A, the euploid blastocysts present 43.00%, and 44.70% were aneuploid blastocysts as well as 12.31% mosaic blastocysts. The euploid blastocysts ratio vitrified on Day 5 were higher than that vitrified on Day 6 and 7 (Fig. 2B). Besides, the blastocysts were quartered with the iDAScore. And results showed that blastocysts with a higher iDAScore contained more euploidy than that with lower iDAScore (Fig. 2C).

**Fig. 3:**
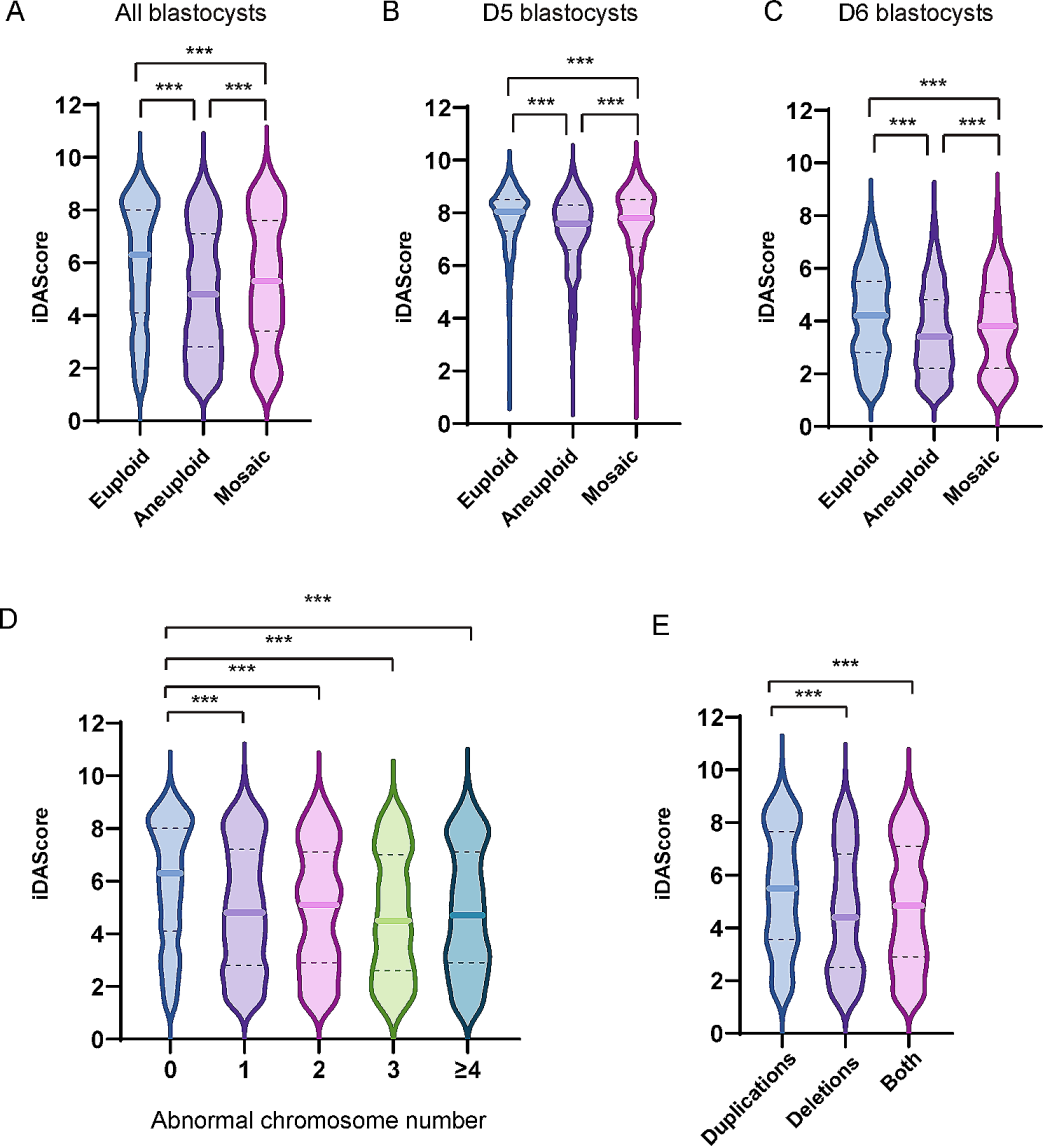
iDAScores of blastocysts with different ploidy. (**A**) iDAScores of all biopsied blastocysts. (**B**) iDAScores of all Day5 biopsied blastocysts. (**C**) iDAScores of all Day6 biopsied blastocysts. (**D**) iDAScores of different abnormal chromosome number. (**E**) iDAScores of different abnormal chromosome type. *** presents *p* < 0.001

Subsequently, the iDAScore was analyzed with different ploidy blastocyst. Results showed that median iDAScore value is 6.3 for euploid blastocyst, 4.8 for aneuploid blastocyst and 5.3 for mosaic blastocyst (Fig. 3A). For Day 5 blastocyst, median iDAScore value is 8.0 for euploid blastocyst, 7.6 for aneuploid blastocyst and 7.8 for mosaic blastocyst (Fig. 3B). For Day 6 blastocyst, median iDAScore value is 4.2 for euploid blastocyst, 3.4 for aneuploid blastocyst and 3.8 for mosaic blastocyst (Fig. 3C).

For the number of abnormal chromosomes, there was a significant difference of iDAScore between normal chromosome and different. However, no significant difference of iDAScore was observed among different chromosome number abnormalities (Fig. 3D).

For chromosome types, a notable dissimilarity of iDAScore was observed in the blastocytes with duplications and deletions, as well as both duplications and deletions. Conversely, no significant dissimilarity was found in the ratio of deletions compared to both duplications and deletions (Fig. 3E).

Table 2 showed the results of multi-variable logistic regression analysis for euploidy prediction. The multivariable logistic regression was adjusted for Garnder criteria, cleavage type, parental chromosome, and length of incubation. The iDAScore was significantly correlated with euploidy prediction in multivariable logistic regression.

**Table 2:** Multi-variable logistic regression analysis for iDAScore on euploid prediction

	Odds ratio	95CI%	*p* value
iDAScore	0.840	0.796–0.887	< 0.001

CI, confidence interval

### Area under the curve (AUC) of euploidy prediction

**Fig. 4:**
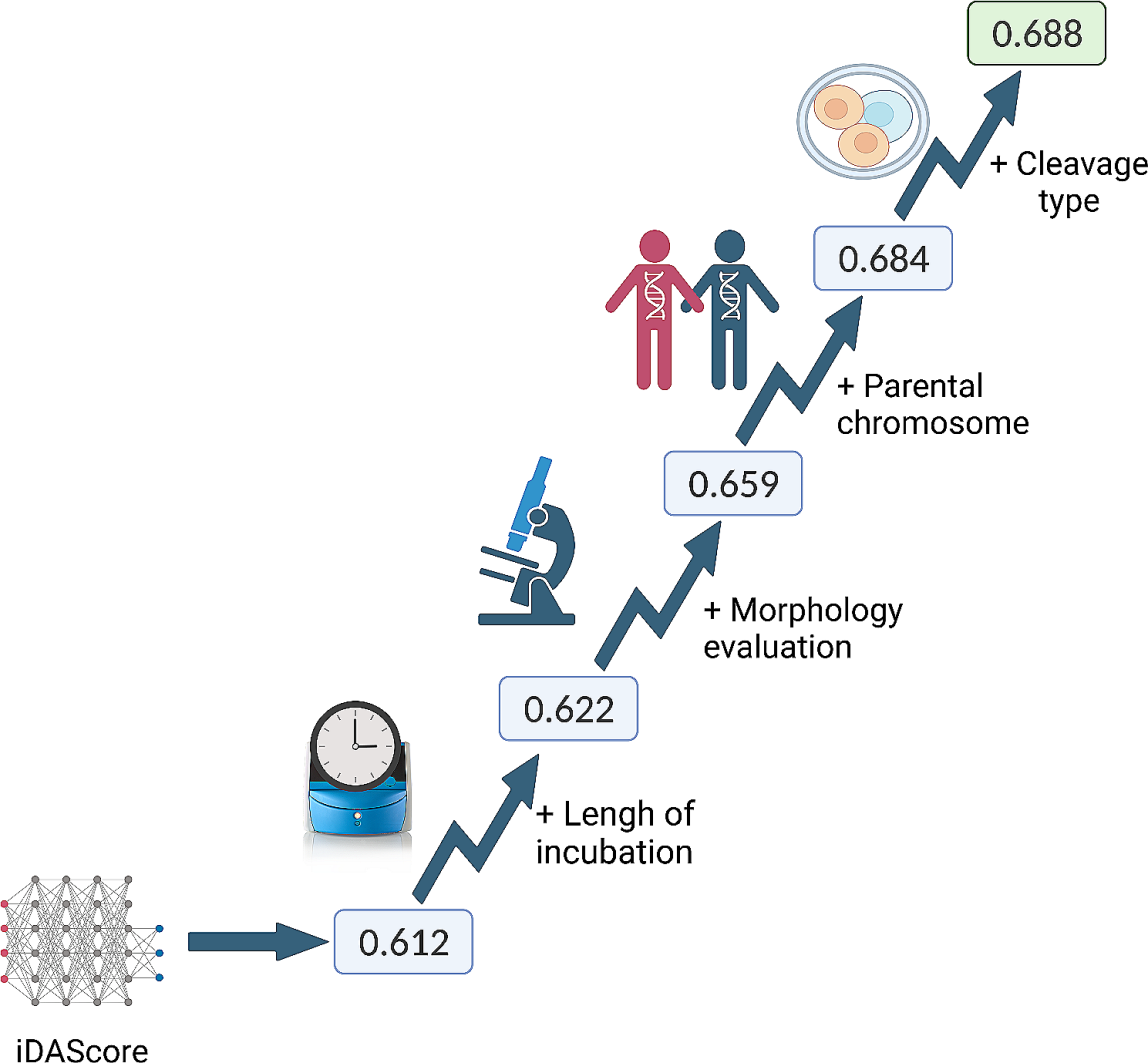
The AUC of iDAScore on euploidy prediction.AUC, Area Under the Curve

The AUC for iDAScore prediction of euploid embryos was 0.612, and when considering the length of blastocyst incubation, the AUC increased to 0.622 (Fig. 4). Furthermore, when incorporating the embryologist’s morphological assessment, the AUC raised to 0.659. When parental chromosome results were added, the AUC further improved to 0.684. Finally, when considering the embryo’s cleavage pattern, the AUC could reach 0.688 (Fig. 4).

## Discussion

In the IVF field, TL system has several benefits over simple, static morphological measurements. By obtaining frequent, microscopic, multiplanar photographs, this enclosed incubation device lessens the need to remove embryos from ideal air culture conditions. These continuous photos allow embryologists to better re-trace embryo development and enable them to make observations without having to take them out of the incubator at a specific point in time. The annotations of an embryo’s morphokinetics, obtained through retrospective study of these photos, can be correlated with outcome factors such as live birth or ploidy status. This makes it possible to choose embryos that show particular development patterns at certain points in time.

Various algorithms and morphokinetic patterns derived from TL data have shown promising results in predicting ploidy [15, 20]. A recent meta-analysis explored morphological and morphokinetic associations with embryo ploidy, revealing that morphokinetic variables such as t8, t9, and tEB were deemed more relevant to ploidy status. Although categorizing aneuploid and euploid embryos with absolute certainty is challenging due to their significant heterogeneity, prioritizing biopsy for certain embryos is conceivable. However, the labeling of particular time points is dependent on individual clinical embryologists, which can lead to some subjective biases.

Currently, AI algorithms use static optical light microscopy images to predict the ploidy state of human embryos [21]. Depending on the dataset, overall accuracy ranged from 60 to 80%, with sensitivity for predicting euploid embryos varied from 75 to 95%. There is a substantial positive association between the fraction of euploid embryos and the genetics AI score in every case, supporting the clinical utility of rating and selecting embryos within a patient cohort that are more likely to be euploid.

KATO et al. investigated the significant correlation between euploidy and the iDAScore, KIDScore Day 5, and Gardner criteria used for blastocyst evaluation [22]. Our findings mirror theirs, demonstrating a substantial correlation between euploidy rates and iDAScore (*p* < 0.001). In contrast, notable differences in KIDScore and Gardner criteria were evident primarily among younger patients. Another study reported AUCs of 0.60 for iDAScore in predicting euploidy, comparable to embryologists’ performance [23]. In our investigation, the combined AUC for euploidy prediction incorporating iDAScore and clinical/embryonic factors reached 0.688. However, in a retrospective simulation analysis, iDAScore v1.0 tended to assign a top-quality ranking to euploid blastocysts in 63% of instances characterized by the presence of one or more euploid and aneuploid blastocysts. Conversely, in situations featuring two or more euploid blastocysts and at least one live birth, iDAScore v1.0 raised questions regarding embryologists’ ranking decisions in 48% of the cases considered. Consequently, iDAScore may serve to objectify embryologists’ assessments.

## Conclusion

This study contributes additional information to strengthen the clinical applicability of iDAScore. The AUC of iDAScore combined with clinical features reached to 0.688. This offers a potential non-invasive and cost-effective alternative for patients without available blastocyst for biopsy or those facing economic constraints. Nonetheless, the accuracy of embryo ploidy remains contingent on the results of NGS analysis. As such, more clinical trials need to be conducted in order to verify the accuracy of iDAScores in predicting embryo ploidy, as well as to provide more data in support of the iDAScore as a predictor of embryo ploidy.

## Data Availability

No datasets were generated or analysed during the current study.

## Abbreviations

AI: Artificial intelligence
AUC: Area Under the Curve
BMI: Body mass index
CI: Confidence interval
CNVs: Copy number variations
COCs: Cumulus-oocyte complexes
COS: Controlled ovarian stimulation
FSH: Follicle-stimulating hormone
Gn: Gonadotropin
HCG: Human chorionic gonadotropin
ICSI: Intracytoplasmic sperm injection
iDAScore: Intelligent data analysis Score
IVF: In in vitro fertilization
MALBACs: Multiple annealing and looping-based amplification cycles
NGS: Next-generation sequencing technology
PGT-A: Preimplantation genetic testing for aneuploidies
TL: Time-lapse
WGA: Whole-genome amplification
